# Prediction of Survival Rate and Chemotherapy Effect by an Immune Score Model in Colorectal Cancer

**DOI:** 10.1155/2022/8219701

**Published:** 2022-04-04

**Authors:** Siyao Liu, Zhengjian Wang, Michael Ntim, Jingrun Han, Xutao Jiang, Chuanfa Fang, Caiming Xu, Jing Zhang

**Affiliations:** ^1^Department of Anesthesiology, Dalian Women and Children's Medical Group, Dalian, Liaoning 116000, China; ^2^Department of General Surgery, The First Affiliated Hospital of Dalian Medical University, Dalian, Liaoning 116000, China; ^3^Department of Physiology, School of Medicine and Dentistry, Kwame Nkrumah University of Science and Technology, Kumasi, Ghana; ^4^Department of Gastroenteric Hernia Surgery, Ganzhou Hospital Affiliated to Nanchang University, Jiangxi 341000, China; ^5^Department of Digestive Endoscopy, Dalian Municipal Central Hospital, Dalian, Liaoning 116000, China

## Abstract

Colorectal cancer is the third most common cancer and the second leading cause of cancer-related deaths. Immune cells in the tumor microenvironment play an important role in the development of tumors. In this study, CIBERSORT was used to estimate the subset of the immune cells using bulk gene expression data (i.e., TCGA, GEO, and cBioPortal databases). 1,087 samples were included in the analysis. The results revealed that among the 22 immune cell subsets that were evaluated, resting and activated NK cells, macrophage M1 and M2, and resting mast cells are associated with significant improvements in patient survival of colorectal cancer. The 15-year survival rates for the training cohort showed 49.1% and 32.5%, respectively, for the low- and high-risk groups. Likewise, the validation and entire cohorts showed 77.3% versus 47.2% and 65.3% versus 46.5%, respectively, for the low- and high-risk groups. Also, the prognostic immune score in predicting the chemotherapy effects showed that the low-risk group had a better survival superiority over the high-risk group, whether patients received chemotherapy or not. The gene set enrichment analysis showed that the low-risk group was highly enriched in pathways or processes related to immune response. The immune checkpoint assessment revealed significantly higher mRNA expressions of CTLA4 in the lower risk group than in the higher risk group. Altogether, this study offers information that could improve the prognosis of colorectal cancer.

## 1. Introduction

Colorectal cancer is one of the major forms of cancer in the alimentary canal. It is the third most common form of tumor and has high morbidity in the world today [1, 2]. It has recently been reported that over a million people develop colorectal cancer every year and mortality is high in developed countries reaching about 45% in recent years [3]. The high incidence of colorectal cancer has been attributed to the changes in people's diet as well as their lifestyle [4–6], but these also affect the prognosis of colorectal cancer. For example, the correlation between obesity and the prognosis of colorectal cancer has always been controversial [7].

The strength of the adaptive immune system has been strongly linked with recurrence as well as survival in colon cancer [8–11]. The role played by the adaptive immune response at the tumor site is pivotal in the balance between tumor invasion and defense against cancer. Many immune cells are associated with the prognosis of colorectal cancer [12]. The presence of immune cells in the tumor microenvironment plays a role in the development of the tumor. It has been reported that tumor-infiltrating immune cell (TIIC) components (type, functional orientation, density, and location) in the solid tumor can convincingly predict the clinical outcome [13, 14]. These have been suspected to be a positive indicator of patient outcomes for a long time [15].

The process of tumor progression demands some level of interaction with tumor cells, microenvironment, and immune system, which act to influence tumor occurrence and development [16]. Research has recently suggested that immune cells serve a momentous role in their function and effect in clinical manifestations of tumors [13, 17]. Much more research has demonstrated that high infiltration of immune cells has been relevant to enhance clinical manifestations and cure rates in colorectal cancer [18, 19].

Therefore, it is of great necessity to establish underlying biomarkers that depend on the whole TIICs' landscape to improve prognosis and prediction and make a diagnosis and give treatment in colorectal cancer patients. The cell type Identification by Estimating Relative Subsets of RNA Transcripts (CIBERSORT), a new calculational method, is used for estimating immune cell subgroups and uses a large body of gene expression data [20]. Here, CIBERSORT is used to quantify 22 TIICs in primary colorectal cancer in patients' data from the TGCA, GEO, and cBioPortal databases. Using single and multiple factor regression analysis, we have built a model based on immune correlation to supplement other methods for forecasting the survival rates and profits from adjuvant chemotherapy (ACT) in colorectal cancer patients. Furthermore, gene set expression analysis (GSEA) was performed to find the function and associated processes of the gene sets.

## 2. Methods

### 2.1. Gene Expression Profiles of Colorectal Cancer

**Figure 1:**
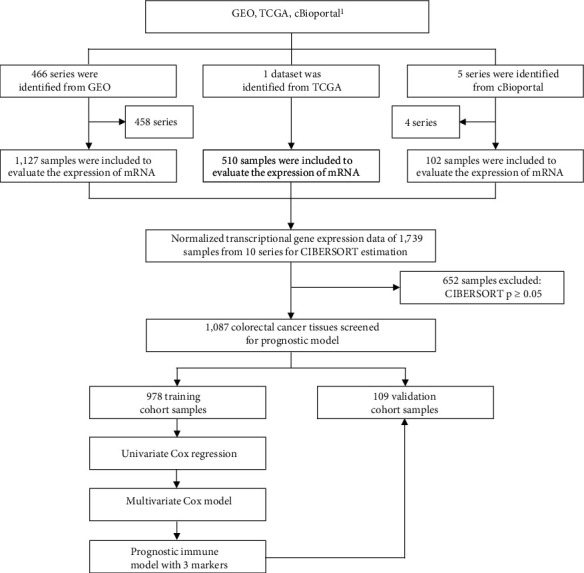
Flow chart of the study design. 1,087 colorectal cancer samples from 466 series, 1 cohort, and 5 series were used to perform CIBERSORT. 652 samples were excluded due to CIBERSORT *p* ≥ 0.05. Three markers were eventually screened to construct a prognostic immune model. The training set (*N* = 978) and the validation set (*N* = 109) were from these public datasets.

The workflow of this study is summarized in Figure 1. The following databases were selected to obtain the gene expression profiles of colorectal cancer tissue: (1) The Cancer Genome Atlas (TCGA, https://portal.gdc.cancer.gov/), (2) Gene Expression Omnibus (GEO, https://www.ncbi.nlm.nih.gov/geo/), and (3) cBioPortal for Cancer Genomics (https://www.cbioportal.org/); and these databases were searched using the keyword “colon cancer”, “colorectal cancer”, or “rectal cancer”. Particularly in GEO, we selected “Homo sapiens” in the Top Organisms filter, “Series” in Entry type filter, and “Expression profiling by array” in the Study type filter. As indicated in Figure 1, 466 series, 1 dataset, and 5 series were identified from GEO, TCGA, and cBioPortal database, respectively. 458 and 4 series were, respectively, excluded from GEO and cBioPortal databases according to the following exclusion criteria: (1) the sample sizes of the series were 30 or fewer; (2) data were obtained from cells, not colorectal tumor tissues; (3) the data were related to microRNA, lncRNA, or DNA, not mRNA; and (4) series for which the survival information of the patients was unavailable. Apart from these criteria for inclusion or exclusion of the series of the databases, the inclusion and exclusion criteria for the patients are as follows: (1) inclusion criteria: all the patients in silico cohort were confirmed as primary CRC and complete clinical records and follow-up information are available and (2) exclusion criteria: CIBERSORT algorithm*p*value > 0.05.

### 2.2. Estimation of Immune Cell Type Fractions

Raw microarray or raw RNA sequencing data which were downloaded from public databases were processed using the MAS5.0 algorithm and normalized using the limma package in R software (version 3.5.2) [21]. To quantify the abundance of 22 TIICs in colorectal tumor specimens, we subsequently performed the CIBERSORT method, an analytical tool, to provide an estimation of the proportions of member cell types in a mixed cell population, using normalized data [20]. The CIBERSORT algorithm was performed online from CIBERSORT web (https://cibersort.stanford.edu/). The following files were required in the website: (1) “LM22.txt” which contains a “signature matrix” of 547 genes (obtained under Menu > Download from CIBERSORT web: https://cibersort.stanford.edu/download.php) [22] and (2) a file containing the normalized mRNA expression data of each sample. The 22 types of infiltration of immune cells inferred by CIBERSORT include B cells, T cells, natural killer cells, macrophages, dendritic cells, eosinophils, and neutrophils. CIBERSORT derives a *p* value for the deconvolution of each sample using Monte Carlo sampling, providing a measure of confidence in the results. At a threshold of *p* < 0.05, 1,087 samples of the inferred fractions of immune cell populations produced by CIBERSORT were considered accurate [23]. The proportions of immune cells were predicted in each dataset separately.

### 2.3. Sampling Method

To improve the precision and accuracy of the prognostic model, 1,087 samples were separated into training and validation sets in a ratio of 9 : 1 using 10-fold CV [24], which was performed using “caret” and “randomForest” packages in R. With a 10-fold CV, a dataset of 1,087 samples was divided into 10 subsets each having 1,087/10 samples. Each of these 10 subsets served in turn as a validation set. A classifier was trained on the remaining 9 × 1, 087/10 samples (the training set), and the trained classifier was then used to classify the 1,087/10 samples in the validation set, generating the prediction error and accuracy. The cross-validation was performed 10 times, and the trained classifier with the highest accuracy score was used to train an ideal prognostic model.

### 2.4. GSEA

The transcriptome data of 170 colorectal tumor samples in GSE17536 from the GEO database were selected for GSEA analysis. GSEA 4.0.3 software (downloaded from https://www.gsea-msigdb.org/gsea/downlodas.jsp) was used to identify GO terms that were enriched between the low- and high-risk groups in the GO database of c5 (c5.all.v6.2.symbols). The significance threshold was set at *p* < 0.05.

### 2.5. Statistical Analysis

The Mann-Whitney *U* test was utilized to compare two groups. The Kruskal-Wallis test was used to compare multiple groups. The univariate and multivariate Cox regression analyses were applied to identify the most significant immune cells to build a prognostic model. The immune cell was considered significant when the *p* value was < 0.05 in the univariate Cox regression analysis. Subsequently, a multivariate Cox regression analysis was applied to optimize the model. The optimal cutoff values were calculated based on the association between survival and cell fraction in the training cohort using the survminer package in R. The Kaplan-Meier analysis and the log-rank test was used to evaluate the correlation between the proportion of immune cells and OS. The prognostic value of the nomogram for 5-, 10-, and 20-year was evaluated by c-index [25]. Results with two-sided *p* < 0.05 were considered to be statistically significant. Statistical analyses were conducted using SPSS version 25 (IBM, New York, USA) and R software (3.5.2).

## 3. Results

### 3.1. The Study Workflow Was Designed

The experiment workflow was first designed as shown in Figure 1. The databases which were selected to acquire the gene expression profiles of the colon or colorectal cancer tissues are as follows: (1) The Cancer Genome Atlas (TCGA, https://portal.gdc.cancer.gov/), (2) Gene Expression Omnibus (GEO, https://www.ncbi.nlm.nih.gov/geo/), and (3) cBioPortal for Cancer Genomics (https://http://www.cbioportal.org/). The terms “Colon cancer”, “colorectal cancer”, or “rectal cancer” were systematically searched in these databases. The entry criteria for the prognostic model were as follows: databases including more than 30 human primary colorectal cancer samples, series provided with overall survival time and survival condition, and the study type being transcriptome profiling. As Figure 1 indicated, 10 studies (1,087 colorectal cancer samples in total) were eventually used to construct the prognostic model.

### 3.2. The Prognostic Immune Score Model Was Established

**Figure 2:**
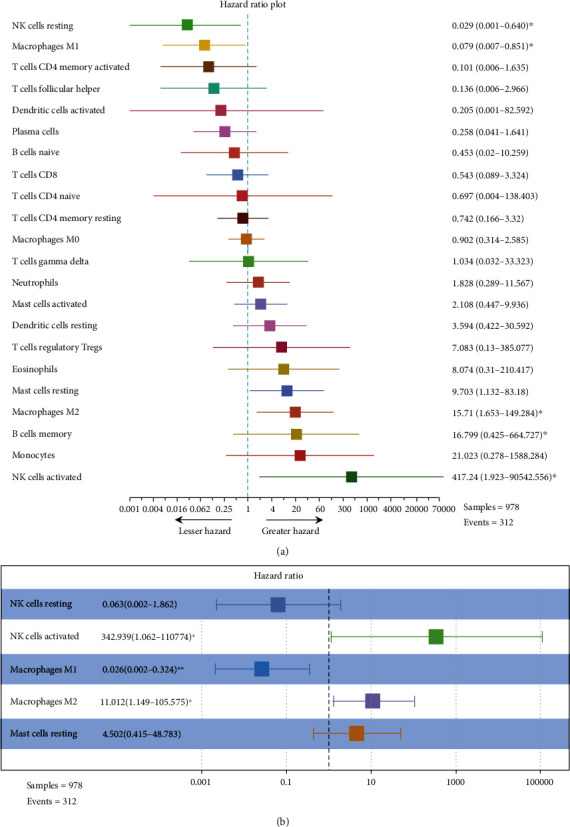
Construction of prognostic immune model in the training cohort. (a) Forest plot of the univariate Cox hazard model for overall survival. Unadjusted HRs were shown with 95% confidence intervals. (b) Optimized model using a multivariate Cox regression analysis which was calculated based on the association between survival and immune cell fraction. This model depicts the specificity and sensitivity of OS prediction based on the immune score of immune cell type: (1) NK cells resting, (2) NK cells activated, (3) macrophage M1, (4) macrophage M2, and (5) mast cells resting. ^∗^Represents *p* < 0.05. ^∗∗^Represents *p* < 0.01.

**Figure 3:**
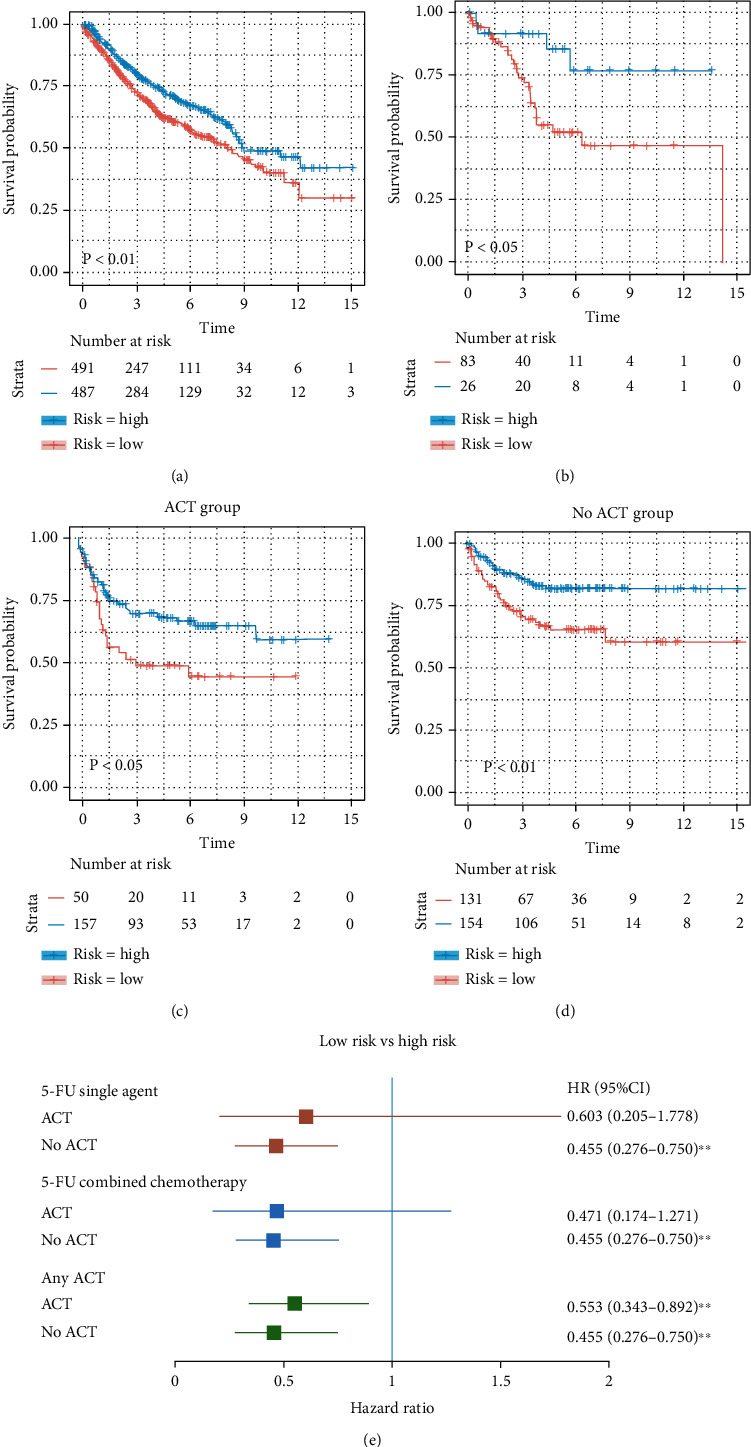
Kaplan-Meier curves of overall survival(OS)of the low- and high-risk patients. (a) Training cohort. (b) Validation cohort. Survival analysis of (c) adjuvant chemotherapy (ACT) and (d) no adjuvant chemotherapy (no-ACT) response among patients with different risk stratification (high or low). (e) Forest plot of the univariate Cox hazard model for OS of the low- and high-risk colorectal cancer patients undergoing different chemotherapy regimens. Unadjusted HRs were shown with 95% confidence intervals.

To estimate the prognostic value of these TIICs, 1,087 samples were randomly divided into the training cohort (*N* = 978) and the validation cohort (*N* = 109) in a ratio of 9 : 1 using the 10-fold cross-validation (10-fold CV) technique. The demographic characteristics of patients can be found in Supplementary Table 1 (Table S1).  Figure 2(a) shows a forest diagram of the relationships between each of the immune cell subgroups and OS in the training cohort. Based on the result of the single factor Cox risk model, resting NK cells (*p* = 0.025), M1 macrophages (*p* = 0.036), resting mast cells (*p* = 0.038), M2 macrophages (*p* = 0.017), and activated NK cells (*p* = 0.028) were significantly correlated to the OS of colon cancer patients. To move forward to identify independent risk factors and compute the prognostic indices, the multiple Cox regression was done (Figure 2(b)). This formula was established by this study for the prognostic immune score model based on the multiple factor Cox regression (risk score = 2.399 × M2 macrophages − 3.660 × M1 macrophages + 5.838 × activated NK cells) (Table S2). The immune score of each sample from the training cohort was computed on the basis of this model. Subsequently, all the samples from the training cohort were divided into the high- or low- risk groups by the cutoff (-0.313), which was acquired by the Optimum Cut points package in R. To assess the OS of the low- and high-risk patients, the Kaplan-Meier curves were performed and significant differences were found in the training cohort (Figure 3(a)). The 15-year survival rates were 49.1% and 32.5%, respectively, for the low- and high-risk groups (hazard ratio (HR) 2.87, 95% confidence interval (95% CI) (1.74-4.73), *p* < 0.0001) (Table 1).

### 3.3. The Prognostic Immune Score Model Was Validated

In order to assess the effect of this prognostic model, the same formula and prognostic immune score model were applied to the validation cohort and the entire cohort. The patients from the validation and entire cohorts were grouped by the cutoff value obtained from the corresponding cohort (validation, -0.086; entire, -0.080). Meanwhile, the Kaplan-Meier curves were performed in the validation cohort (Figure 3(b)) and the entire cohort (Figure S1). The 15-year survival rates were 77.3% and 47.2%, respectively, for the low- and high-risk groups (HR 9.30, 95% CI (1.04-82.86), *p* = 0.046) in the validation cohort (Table 2) and 65.3% and 46.5%, respectively, for the low- and high-risk groups (HR 3.01, 95% CI (1.86-4.89), *p* < 0.0001) in the entire cohort (Table 1).

### 3.4. Chemotherapy Response Was Predicted by the Prognostic Immune Score Model

Neoadjuvant chemotherapy (neo-ACT), as well as adjuvant chemotherapy (ACT), has been reported to be related to immune infiltration [26]. Further evaluation was done to find whether the application of chemotherapy (CT) would influence the prognosis of colorectal cancer. The information regarding the administration of neo-ACT or ACT was collected from the GEO database. The detailed information on adjuvant chemotherapy was documented in the GSE39582 dataset. In order to evaluate the relationship between the immune score and response to chemotherapy, the formula was applied. The patients from the GSE39582 cohort were divided into the low- and high-risk groups by the cutoff value (0.037). The survival advantage for the low-risk group was evident, regardless of whether they received chemotherapy or not (Figures 3(c) and 3(d)). More importantly, the effect of 5-FU as a single agent, combined chemotherapy, and any adjuvant chemotherapy (ACT) was determined. The hazard ratio for patients in the low-risk group was significantly lower in patients who underwent ACT except for the 5-FU chemotherapy regime (Figure 3(e), *p* < 0.01).

### 3.5. The Prognostic Immune Score Model Was Improved by Nomogram

**Figure 4:**
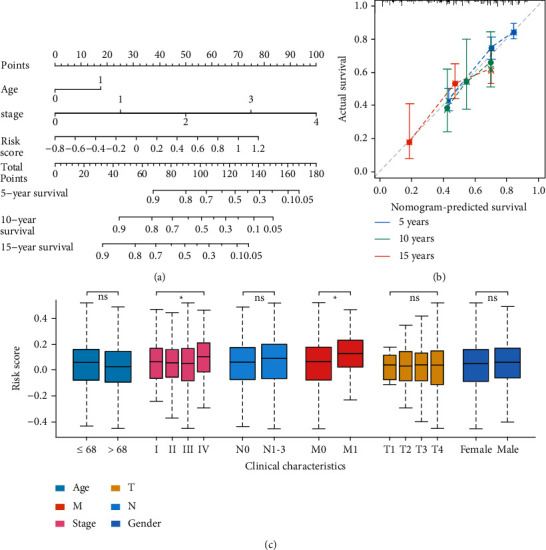
Construction of the nomogram system. (a) Nomogram predicting 5-, 10-, and 15-year overall survival for colorectal cancer patients in the training cohort based on immune score and other clinicopathological parameters such as age and stage. (b) The calibration curves of nomograms between predicted and observed 5-, 10-, and 15-year OS in the training cohort. The dashed line at an angle of 45° represents the perfect prediction of the nomogram. (c) Stratified analysis of clinical characteristics for the immune score of the immune prognostic model for the training cohort.

To select independent clinicopathological prognostic factors for the OS, the univariable Cox regression analysis was performed, and the results showed that age, tumor grade, tumor-node-metastasis (TNM) stage, and the risk score were significantly related to the OS (Table 1). Subsequently, the multivariable Cox regression analysis was performed, and it showed that risk score, age, and TNM stage were the independent prognostic factors for the OS (Table 2). In order to create a quantitative method to predict the probability of OS, we integrated the immune score and independent clinicopathological prognostic factors including age and TNM stage to construct a nomogram (Figure 4(a)).

To evaluate the predictive value of the nomogram, we compared Harrell's concordance index (C-index) of the nomogram with standard TNM staging in the training cohort, the validation cohort, and the entire set. As shown in Table 3, the nomogram system improved the prognostic model of colorectal cancer in the training, validation, and entire set. The calibration plots showed that the predicted 5-, 10-, and 15-year survival probabilities of the nomogram performed well in the training cohort (Figure 4(b)).

### 3.6. The Clinical Covariates of Patients Correlated with the Prognostic Immune Score

The correlation between the prognostic immune score with clinical covariates was further analyzed in the training and validation sets. The TNM stage (*p* < 0.05) and M category (*p* < 0.05) were significantly related to the immune score (Figure 4(c)) in the training set. In the validation cohort, only the M category (*p* < 0.05) was significantly related to the immune score (Figure S2).

### 3.7. Differential Expression of the Genes Associated with Immune Checkpoint Was Predicted by the Prognostic Immune Score Model

**Figure 5:**
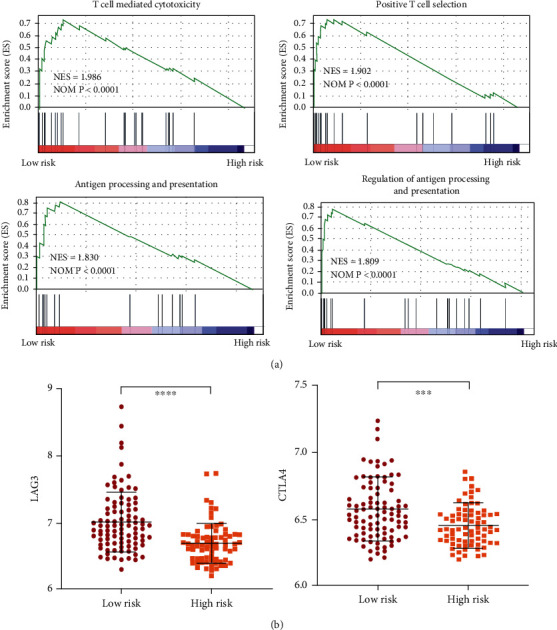
Bioinformatics analysis of the characteristics and signal pathways among patients with different risk groups. (a) Gene set enrichment analysis (GSEA) for immune system pathways and processes correlated with immune score values in the GSE17536 from the GEO database. NES: normalized enrichment score; NOM *p*: nominal *p* value. (b) CTLA4 mRNA expression levels between the low- and high-risk groups in the GSE17536 from the GEO database.

The immune score of 170 colorectal tumor samples from GSE17536 was determined by the prognostic immune formula. All the samples were classified into the low-risk and high-risk groups by the cutoff (0.063). The gene set enrichment analysis (GSEA) showed that the low-risk group was highly enriched with activation of T cell-mediated cytotoxicity, positive T cell selection, antigen processing, and regulation of antigen processing and presentation (Figure 5(a)). These four had normalized enrichment scores (NES) of 1.986, 1.902, 1.830, and 1.809, respectively, and showed their significant enrichment in the low-risk group.

Immune checkpoint blockade with immunotherapies, such as CTLA-4, has been thought to be promising approaches to treat a variety of malignancies. Thus, the expression of several key immune checkpoint regulators, as well as inflammatory mediators, was determined. As shown in Figure 5(b), CTLA-4 and LAG3 were significantly higher in the low-risk groups with *p* < 0.0001 and *p* < 0.001, respectively, for colorectal cancer patients in GSE17536 from the GEO database.

## 4. Discussion

The tumor microenvironment is very critical in determining the progression of cancer and has been widely related to cancer diagnosis and prognosis. The tumor microenvironment is usually composed of stroma cells, cytokines, chemokines, and the cancer cells themselves [27].

Many different types of cancer are infiltrated with TIICs which could be of different subpopulations in various patients. Many immune-related molecules have also been implicated in the progression of various cancers. A study has shown that Cytokeratin 18 has a certain correlation with tumor progression in neoadjuvant chemotherapy for breast cancer [28]. Low molecular weight heparin has been shown to exert antitumor properties by modulating immunity in patients with esophageal cancer [29]. At the same time, some literature has confirmed that Phosphatidylinositol 3-kinase/AKT/Mammalian Target of Rapamycin (PI3K/AKT/mTOR) can be used as an immunotherapy target for esophageal cancer by affecting the expression of microRNA [30]. Fanipakdel et al. have identified melanoma-associated antigen A1 in lung cancer patients as a new immunotherapy target [31]. The Wnt/*β*-catenin pathway has also been demonstrated as an immune target for pancreatic cancer therapy [32]. Our earlier studies have found that the immune scoring model based on immune infiltrating cells has a good predictive effect in evaluating the prognosis and chemotherapy effect of patients with breast cancer [33]. However, there are few studies on the diagnosis and prognosis evaluation of patients with colorectal cancer, which has a good clinical research value. In this research, the assessment of the TIICs using CIBERSORT on the 1087 samples showed that resting and activated NK cells, M1 and M2 macrophages, and activated mast cells were significantly related to the OS of colon cancer patients. Independent studies have found infiltration of macrophages and NK cells in patients with colon cancer [34, 35]. Some of these immune cells have been related to early colon cancer promotion and have contributed to the resistance to chemotherapy for colon cancer patients. Presently, effective biomarkers that help in predicting the prognosis of colon cancer are still under investigation all over the world [36].

Existing guidelines have proposed the consideration of ACT for patients with poorly differentiated histology, T4 stage, lymphovascular invasion, or perineural invasion [37]. This regimen has been shown to be protective in the treatment of tumor prognosis [38]. However, numerous studies have not provided compelling evidence of ACT improving survival in patients with high-risk colon cancer. FU-based adjuvant chemotherapy has been proved not to be beneficial to all patients with colorectal cancer [39, 40]. The effect of miRNAs in the modulation of 5-FU tolerance has been largely evaluated in colorectal cancer (CRC) cells. Upregulation of miR-15b-5p has strengthened 5-FU-associated cell apoptosis and improved the cell response to 5-FU both in vitro and in animal models [41]. A study by Booth et al. concluded that ACT does not have any association with the survival among stage II colon cancer patients and those who have been classified as high risk for the disease [42]. Another study, in contrast, had reported earlier that ACT was associated with improved survival [43]. The results of this current study are consistent with the statement earlier made and also with the results from our analysis in this study. M2 macrophages are important in the release of circulating tumor cells (CTCs). Studies have proved that M2 TAMs tend to promote directional migration in tumor cells' vessels and invasion by the paracrine loop of tumor-derived CSF-1 and TAM-derived EGF/EGF-like ligands [44, 45], secrete osteonectin [46], Cathepsin [47], and TGF-Beta [48]. Numerous reports have found tumor-associated M2 macrophages to predict worse outcomes than M1 macrophages [49].

The GSEA is a computational method to explore if a given set of genes are significantly involved in some pathways. In this study, the GSEA depicted that the low-risk colon tumor samples from the dataset were enriched with processes involved in immune responses or entails changes in the tumor microenvironments. Hence, the results in this study reveal many specific biological processes involved in the immune cell microenvironment.

CTLA-4 negatively regulates immune responses and has been reported as critical for controlling TIICs [50]. Recently, abnormal expression of CTLA-4 has been reported in numerous tumors and is believed to contribute to the initiation and progression of cancer [51, 52]. As reported in our study, the expression of CTLA-4 in the high-risk group was relatively low compared to the low-risk group. This observation is not out of place as a meta-analysis study has been thought to link CTLA-4 polymorphisms to the development of digestive system cancer [53]. Other studies have reported an increased expression in CTLA-4, which was related to worse outcomes for cancer, and this is contrary to the results of this present study. These differences in the reports call for the need to validate this observation in large clinical samples in future experiments. However, antibodies generated against CTLA-4 have been proposed as effective in the treatment of a variety of cancers [54]. In a meta-analysis, LAG3 was reported to be associated with improved overall survival [55]. Their effects were somewhat consistent in different tumor types. In many cancers, the combination of LAG3 and PD-1 inhibition has been synergic [56]. In some melanoma patients, CTLA-4 inhibition has been found to elicit an increase in the frequency of LAG3+ TILs [57]. So far, no study has reported such in colorectal cancers. Further studies are needed to establish the mechanisms of CTLA-4 and LAG3 in patients with colorectal cancer. Upregulation of CTLA-4 and LAG3 molecules can initiate a negative feedback mechanism that creates an active immune environment in an inflamed tumor and can improve prognosis [58]. The observation in this current study showed this increased expression of CTLA-4 and LAG3 in the low-risk group, a situation that needs further research to establish if this observation is real.

Even though some studies have used immune cell infiltration to establish a prognostic model, such studies did not validate with any external cohort from the cancer databases. This study on the other hand used data from the external database, and there is the need to compare these database results to clinically collected samples that serve as the test cohort. A meta-analysis is therefore needed to pool together all these studies to assess their overall outcome on the diagnosis and prognosis of colorectal cancer. We collected about 1087 samples from different databases, whose data volume is large enough and persuasive. The immune scoring model was repeatedly verified, and the relationship between it and colorectal cancer was verified by single factor and multifactor regression models, and it was combined with clinical indicators and chemotherapy, which has great clinical application value. These advantages offer a comparative advantage over the other published work in offering a better diagnosis and prognosis of CRC.

In summary, this study analyzed the critical immune infiltrates using CIBERSORT and used them to assess their prognostic performance in colon cancer together with other factors such as age, grade, and TNM stage. They were used in predicting the overall survival in 5, 10, and 15 years. The effect on the survival of patients undergoing adjuvant chemotherapy and no adjuvant chemotherapy treatment has also been reported. All these results will help to improve the diagnosis and enhance the prognosis of colorectal cancer.

## Figures and Tables

**Table 1:** Results of the univariable Cox regression analysis.

Variables	Training cohort		Validation cohort	
	HR (95% CI)	*p* value	HR (95% CI)	*p* value
Risk score	2.87 (1.74-4.73)	<0.0001	9.30 (1.04-82.86)	0.046
Age (>60 vs. ≤60)	1.54 (1.22-1.95)	0.0003	1 (0.48-2.09)	0.992
Gender (male vs. female)	1.26 (1-1.59)	0.052	0.90 (0.43-1.89)	0.780
Differentiation (vs. high)				
Middle	2.45 (0.9-6.71)	0.081	/	0.25
Low	4.28 (1.46-12.62)	0.008	3.26 (1.08-3.26)	0.035
Stage (vs. stage I)				
II	1.54 (0.86-2.74)	0.146	1.96 (0.25-15.50)	0.523
III	2.13 (1.19-3.80)	0.010	3.42 (0.44-26.50)	0.239
IV	9.57 (5.31-17.23)	<0.0001	10.04 (1.26-79.70)	**0.029**

**Table 2:** Multivariable Cox regression analysis.

Multivariable cox regression analysis				
Variables	Training cohort		Validation cohort	
	HR (95% CI)	*p* value	HR (95% CI)	*p* value
Risk score	8.80 (2.30-33.72)	0.002	18.86 (1.37-260.40)	0.03
Age (>60 vs. ≤60)	1.89 (1.16-3.06)	0.01	0.93 (0.40-2.16)	0.87
Gender (male vs. female)	1.16 (0.72-1.86)	0.55	0.62 (0.27-1.43)	0.26
Differentiation (vs. high)				
Middle	0.98 (0.34-2.79)	0.97	1.07 (0.60-1.91)	NA
Low	2.64 (0.84-8.26)	0.10	1.26 (0.88-1.81)	NA
Stage (vs. stage I)				
II	4.29 (0.55-33.18)	0.16	1.72 (0.21-14.18)	0.62
III	8.35 (1.10-63.37)	0.04	2.24 (0.26-19.23)	0.46
IV	40.58 (5.35-307.72)	0.0003	18.86 (1.37-260.40)	0.04

**Table 3:** C-index of TNM stage and nomogram model.

Cohort	C-index (95% CI)	
	TNM stage	Nomogram
Training	0.663 (0.643-0.684)	0.683 (0.667-0.670)
Validation	0.676 (0.629-0.723)	0.736 (0.690-0.782)
Entire	0.682 (0.667-0.697)	0.750 (0.736-0.765)

## Data Availability

The datasets used and/or analyzed during the current study are available from the corresponding author on reasonable request.

## Supplementary Materials

Supplementary 1 Figure S1: Kaplan-Meier curves of overall survival(OS)of the low- and high-risk patients in the entire cohort. Figure S2: stratified analysis of clinical characteristics for the immune score of the immune prognostic model in the validation cohort.

Supplementary 2 Table S1: the demographic characteristics of patients. Table S2: the formula of colon cancer immune infiltration model.
