# Supplementary figures and images for: Prediction of Survival Rate and Chemotherapy Effect by an Immune Score Model in Colorectal Cancer
Source: Biomed Res Int. 2022 Apr 4;2022:8219701. doi: 10.1155/2022/8219701 (PMC9006078; doi:10.1155/2022/8219701)

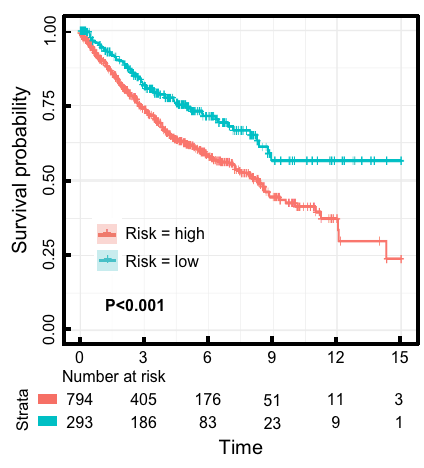

Supplement: Supplementary 1 — Figure S1: Kaplan-Meier curves of overall survival(OS)of the low- and high-risk patients in the entire cohort. Figure S2: stratified analysis of clinical characteristics for the immune score of the immune prognostic model in the validation cohort. [file 8219701.f1.zip › Fig.S1.png]

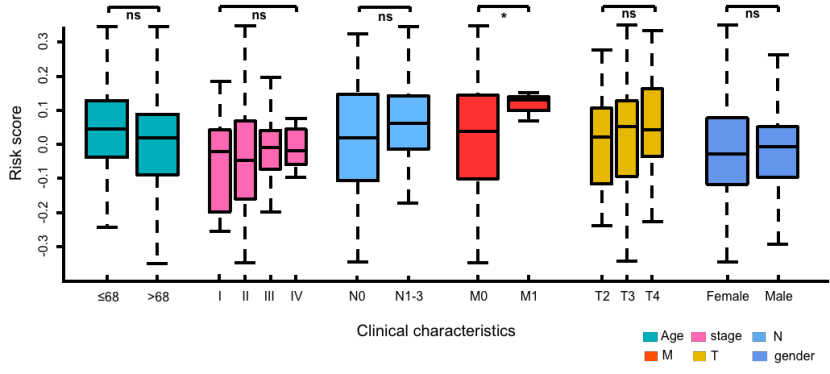

Supplement: Supplementary 1 — Figure S1: Kaplan-Meier curves of overall survival(OS)of the low- and high-risk patients in the entire cohort. Figure S2: stratified analysis of clinical characteristics for the immune score of the immune prognostic model in the validation cohort. [file 8219701.f1.zip › Fig.S2.png]
