# Supplementary material for: Prediction of Survival Rate and Chemotherapy Effect by an Immune Score Model in Colorectal Cancer
Source: Biomed Res Int. 2022 Apr 4;2022:8219701. doi: 10.1155/2022/8219701 (PMC9006078; doi:10.1155/2022/8219701)
Supplement: Supplementary 2 — Table S1: the demographic characteristics of patients. Table S2: the formula of colon cancer immune infiltration model. [file 8219701.f2.docx]

**Table S1. Demographic characteristics of patients**

| **characteristic** |  |  |  | **Traning Cohort(n=978)**  **n (%)** | | **Validation Cohort(n=109)**  **n (%)** | | |
| --- | --- | --- | --- | --- | --- | --- | --- | --- |
| **Age(years)** | **Median(range)** | | | 68(21-96) |  | | 66(24-93） |  |
|  | **Unknown** | | | 57(5.8) |  | | 8(7.4) | - |
| **Gender** | **Male** | | | 485(49.6) |  | | 65(59.6) |  |
|  | **Female** | | | 443(45.3) |  | | 41(37.6) |  |
|  | **Unknown** | | | 50(5.1) |  | | 3(1.8) |  |
| **TNM** | **T** | **N** | **M** |  |  | |  |  |
|  | 1 | 0 | 0 | 10(1.0) |  | | - |  |
|  |  |  | 1 | 1(0.1) |  | | - |  |
|  |  | 1 | 0 | 2(0.2) |  | | - |  |
|  | 2 | 0 | 0 | 26(2.7) |  | | 6(5.5) |  |
|  |  |  | NA | 1(0.1) |  | | / |  |
|  |  | 1 | 0 | 9(0.9) |  | | / |  |
|  |  |  | NA | 1(0.1) |  | | / |  |
|  |  | 2 | 0 | 4(0.4) |  | | / |  |
|  |  |  | NA | 1(0.1) |  | | / |  |
|  |  | 3 | 0 | 1(0.1) |  | | / |  |
|  | 3 | 0 | 0 | 172(17.7) |  | | 24(22.2) |  |
|  |  |  | 1 | 4(0.4) |  | | 2(1.8) |  |
|  |  |  | NA | 12(1.3) |  | | / |  |
|  |  | 1 | 0 | 89(9.1) |  | | 8(7.3) |  |
|  |  |  | 1 | 12(1.3) |  | | / |  |
|  |  |  | NA | 7(0.7) |  | | / |  |
|  |  | 2 | 0 | 48(4.9) |  | | 5(4.6) |  |
|  |  |  | 1 | 11(1.1) |  | | / |  |
|  |  |  | NA | 3(0.3) |  | | 1(0.9) |  |
|  |  | 3 | 0 | 3(0.3) |  | | / |  |
|  |  | NA | 0 | 3(0.3) |  | | / |  |
|  |  |  | 1 | 1(0.1) |  | | / |  |
|  | 4 | 0 | 0 | 43(4.4) |  | | 6(5.5) |  |
|  |  |  | 1 | 3(0.3) |  | | / |  |
|  |  |  | NA | 1(0.1) |  | | 1(0.9) |  |
|  |  | 1 | 0 | 13(1.3) |  | | 2(1.8) |  |
|  |  |  | 1 | 7(0.7) |  | | / |  |
|  |  |  | NA | 1(0.1) |  | | / |  |
|  |  | 2 | 0 | 17(1.7) |  | | 1(0.9) |  |
|  |  |  | 1 | 10(1.0) |  | | 1(0.9) |  |
|  |  |  | NA | 2(0.2) |  | | / |  |
|  |  | 3 | 1 | 1(0.1) |  | | / |  |
|  |  | NA | 0 | 2(0.2) |  | | 1(0.9) |  |
|  | NA | 0 | 0 | 2(0.2) |  | | 1(0.9) |  |
|  |  | NA | NA | 455(46.5) |  | | 50(45.9) |  |
| **Stage** | 1 | | | 64(6.5) |  | | 8(7.3) |  |
|  | 2 | | | 347(35.5) |  | | 39(35.8) |  |
|  | 3 | | | 291(29.8) |  | | 29(26.6) |  |
|  | 4 | | | 113(11.6) |  | | 13(11.9) |  |
|  | NA | | | 163(16.6) |  | | 20(18.4) |  |
| **Risk score** | ≥-0.313 | | | 920(94.1) |  | | 106(97.2) |  |
|  | ＜-0.313 | | | 58(5.9) |  | | 3(2.8) |  |

NA：unknow

**Table S2. Formula of colon cancer immune infiltration model**

| **Immune cell marker** | **Coefficients** | **SE** | ***P*-Value** |
| --- | --- | --- | --- |
| Macrophages M1 | -3.660 | 1.292 | 0.005 |
| Macrophages M2 | 2.399 | 1.153 | 0.038 |
| NK cells activated | 5.838 | 2.948 | 0.048 |
